# Contribution of diet and major depression to incidence of acute myocardial infarction (AMI)

**DOI:** 10.1186/1476-511X-9-133

**Published:** 2010-11-18

**Authors:** Teymoor Yary, Kourosh Soleimannejad, Firdaus Abd Rahim, Mirnalini Kandiah, Sanaz Aazami, Seyedehozma Jafar Poor, Wong Teck Wee, Golnaz Aazami

**Affiliations:** 1 Department of Nutrition and Dietetics, Faculty of Medicine and Health Sciences, University Putra Malaysia, 43400 UPM, Serdang, Selangor, Malaysia; 2 Department of Medicine, Faculty of Medicine and Health Sciences, Ilam Medical University, 6939177143, Banganjab, Ilam, Iran; 3 Department of Medicine, Faculty of Medicine and Health Sciences, University Putra Malaysia, 43400 UPM, Serdang, Selangor, Malaysia; 4 Department of Community Health, Faculty of Health, Tehran Medical Branch, Islamic Azad University, Zargandeh, 19168, Tehran, Iran

## Abstract

**Background:**

Despite significant improvements in the treatment of coronary heart disease (CHD), it is still a major cause of mortality and morbidity among the Iranian population. Epidemiological studies have documented that risk factors including smoking and the biochemical profile are responsible for the development of acute myocardial infarction (AMI). Psychological factors have been discussed as potential risk factors for coronary heart disease. Among emotional factors, depression correlates with coronary heart disease, particularly myocardial infarction.

**Methods:**

This case-control study was conducted on 120 cases (69 males and 51 females) of acute myocardial infarction (AMI) and 120 controls, with a mean age of 62.48 ± 15.39 years. Cases and controls were matched by age, residence and sex.

**Results:**

The results revealed that severe depression was independently associated with the risk of AMI (P = 0.025, OR = 2.6, 95% CI 1.1-5.8). The analysis of variables indicated that risk factors for developing depression were unmarried, low levels of polyunsaturated fatty acids (PUFAs), total dietary fiber (TDF) and carbohydrates. The levels of these dietary factors were lowest in severely depressed patients compared to those categorised as moderate or mild cases. Furthermore, severely depressed subjects were associated with higher levels of total cholesterol, high systolic blood pressure (SBP) and WHR. Age, income, a family history of coronary heart disease, education level, sex, employment and smoking were not associated with severe depression.

**Conclusion:**

The present study demonstrated that severe depression symptoms are independent risk factors for AMI. Furthermore, severe depression was associated with an unhealthy diet and AMI risk factors.

## Background

Despite significant improvements in the treatment of coronary heart disease (CHD), it is still a major cause of mortality and morbidity among the Iranian population [1]. Epidemiological studies have documented that risk factors including smoking and the biochemical profile are responsible for the development of acute myocardial infarction (AMI) [2-4].

Psychological factors have been discussed as potential risk factors for coronary heart disease [5]. Among emotional factors, depression correlates with coronary heart disease, particularly myocardial infarction [6]. Major depression has been associated with approximately 20% of patients newly diagnosed with CHD, and with patients who have suffered but recovered from myocardial infarction (MI) [7]. Depression has been related to various heart disease risk factors such as an unhealthy diet [8,9], anthropometric parameters, marital dissatisfaction [10], and hypertension [11,12]. Many MI patients say they continue to smoke and persist with an unhealthy diet because of depression, despite these being well-known risk factors for MI [13]. While depression appears to be a risk factor for myocardial infarction, it is not clear whether it should be considered as an independent risk factor for this disease [14].

The aim of the present study was to investigate the association of severe depression and AMI risk factors such as diet. In addition, we aimed to assess, after adjusting for other variables, whether severe depression is an independent risk factor for acute myocardial infarction (AMI).

## Materials and methods

This study was approved by the Ethics Committee of the University Putra Malaysia and informed consent was obtained from all participants before enrolment in the study.

### Study subjects and study design

This case-control study was conducted on 120 cases (69 males and 51 females) of acute myocardial infarction (AMI) and 120 controls, with a mean age of 62.48 ± 15.39 years. AMI was defined by clinical criteria, electrocardiographic criteria and biochemical markers. Cases of AMI were recruited from individuals who suffered from chest pain and were admitted to the emergency department of Mostafa hospital. Control cases comprised 120 individuals who received routine health checkups at the same hospital. Cases and controls were matched by age, residence and sex. Exclusion criteria included notable chronic medical illness (e.g. untreated hyperthyroidism or hyperthyroidism, renal disease or malignant disease, and pregnancy) as these conditions could change an individual's lifestyle or modify the risk factors for AMI. Cases and controls that had a history of mental illness and received anti-depressants or other psychiatric drugs were excluded. Furthermore, controls who had positive electrocardiographic results were excluded.

### Determination of dietary variables

A semi-quantitative food frequency questionnaire (FFQ) developed by Willet [15] and validated extensively in several multi-ethnic populations was used to collect dietary information. The FFQ was used to measure long-term (over the previous 12 months) intake of food. The list of foods in the FFQ was modified to reflect the traditional diet of the Ilam province. The FFQ data were obtained during a face-to-face interview with subjects. Nutrition IV software version 3.5.2 was used for analysis of nutrient intake. The database included a standard reference, which contained several different foods and nutrients, and values for nutrients such as carbohydrates and fats.

### Determination of biomarkers

The venous blood of participants was collected by a nurse at the Mostafa Hospital between 07.00 and 09.00 hours, 12-14 hours after an overnight fast, and centrifuged between 30 to 40 minutes after collection; blood samples from AMI patients were collected within 24 hours of fasting. Fasting levels of serum total cholesterol (TC), triglycerides (TG), low density lipoproteins (LDL), high density lipoproteins (HDL) and fasting blood sugar (FBS) were determined using standard laboratory methods at the Mostafa Hospital, Ilam. Fasting blood sugar (FBS) and lipid profiles were measured using the Pars Azmoon Kit, which has been described in earlier studies [16,17].

### Blood pressure

A standard manometer was used by a nurse or physician to measure the blood pressure (BP) of participants. The first BP readings were taken with individuals in the sitting position and after five minutes rest. Two separate readings for the blood pressure of each participant were taken with a 10-minute interval between them. The averages of these readings were recorded and used for final data analysis.

### Depression questionnaire

The present study used the Centre for Epidemiological Studies Depression Scale (CES-D) to measure depression. The CES-D was translated into Persian by the researcher and used to obtain the cut off point for this study.

The CES-D scale was originally developed to measure depression. The Centre for Epidemiological Studies developed the questionnaire with a 20-interview format or self-report items for the purpose of measuring current levels of symptoms of depression. These items included appetite loss, sleep disorders, sadness, crying, feelings of loneliness and fear. The CES-D 4-point scale is: 0 = rarely or none of the time (less than 1 day); 1 = some or a little of the time (1-2 days); 2 = occasionally or a moderate amount of time (3-4 days); 3 = most or all of the time (5-7 days).

Four items (4, 8, 12, and 16) in this questionnaire were reversed. The total depression score was calculated by summing up the item scores. Generally, average scores were between 0 and 60, and higher scores indicated more depressive symptoms. For the high depressive symptoms category a cut-off score of 21 was used as a determinant of severe depression. The baseline characteristics for depression status were established in the controls, and depression in positive cases was assessed after three months.

### Statistical analysis

SPSS for Windows version 18 was used for statistical analyses. Cases and controls were compared using parametric and non-parametric tests including chi-square test, independent sample t-test and the Mann-Whitney U test. For final analysis, binary unconditional logistic regression analysis was conducted to identify severe depression as an independent risk factor for AMI in the case-control model.

## Results

The mean age of the participants was 62.48 ± 15.39 years and Table 1 presents the baseline variable data for the 240 participants (120 cases and 120 controls). Univariate analysis of continuous variables indicated that risk factors for developing AMI were low TDF, WHR, FBS, TC, TG, LDL, SBP, DBP, MUFA and PUFA. A family history of coronary artery disease and smoking increased the risk of developing AMI. Most of the subjects enrolled in the study had not been educated to the level of higher secondary school or beyond and most were also married. There were no significant differences in terms of education level and marital status between the experimental and control groups.

**Table 1:** Baseline variables for cases and controls

Variables	Cases (n = 120) Mean ± SD	Controls (n = 120) Mean ± SD	p
Family history of CHD,%	36.7	10.8	0.000
Current smoker, %	22.5	8.3	0.002
Former smoker, %	12.5	7.5	0.197
Married, %	78.3	91.7	0.004
Education ≥ higher secondary school, %	22.5	30.0	0.187
Employed, %	51.7	71.7	0.001
SBP (mm/Hg)	150.05 ± 14.92	139.17 ± 19.78	0.000
Income ($)	409.25 ± 194.15	395.41 ± 199.61	0.587
DBP (mm/Hg)	98.76 ± 11.28	91.65 ± 13.04	0.000
FBS (mg/dl)	129.89 ± 75.58	100.60 ± 32.23	0.000
TC (mg/dl)	217.45 ± 30.22	184.65 ± 36.19	0.000
TG (mg/dl)	177.62 ± 97.53	143.64 ± 41.68	0.001
LDL-C (mg/dl)	151.84 ± 24.55	135.55 ± 23.63	0.000
HDL-C(mg/dl)	62.70 ± 12.77	59.74 ± 12.18	0.109
TDF (g/day)	12.89 ± 5.71	17.63 ± 6.36	0.000
Carbohydrates (g/day)	219.05 ± 56.54	259.12 ± 59.27	0.000
SFA (g/day)	17.32 ± 5.83	17.86 ± 6.36	0.488
MUFA (g/day)	16.51 ± 5.92	19.20 ± 6.20	0.001
PUFA (g/day)	13.61 ± 10.14	16.93 ± 9.91	0.011
WHR	1.04 ± 0.18	0.97 ± 0.20	0.006
BMI (kg/m^2^)	28.70 ± 5.36	27.96 ± 5.24	0.280

The participants were divided according to the absence or presence of severe depression symptoms, calculated from the total depression questionnaire. The prevalences of severe depression in the case and control groups were 36.7% and 15.8%, respectively. The present study demonstrated that severe depression was significantly associated with unmarried subjects. Further analyses demonstrated no significant differences between the groups in terms of relationships between severe depression, a family history of coronary heart disease, education level, sex, employment and smoking (Table 2).

**Table 2:** Categorical variables based on depression levels

Variables	Severe Depression		
	Absent (N = 177)	Present (N = 63)	p
Case group/control group, %	63.3/84.2	36.7/15.8	0.000
Married, %	89.3	73.0	0.002
Education ≥ higher secondary school, %	27.7	22.2	0.398
Sex, %			
Female	44.1	38.1	0.410
Male	55.9	61.9	
Employed, %	62.1	60.3	0.798
Family history of CHD, %	23.7	23.8	0.990
Current smoker, %	15.3	15.9	0.907
Former smoker,%	10.2	9.5	0.883

The analysis of variables indicated that risk factors for developing depression were low levels of polyunsaturated fatty acids (PUFAs), total dietary fiber (TDF) and carbohydrates. The levels of these dietary factors were lowest in severely depressed patients compared to those categorised as moderate or mild cases. Furthermore, severely depressed subjects were associated with higher levels of total cholesterol, high systolic blood pressure (SBP) and WHR. Age and income were not associated with severe depression (Table 3).

**Table 3:** Continuous variables according to depression categories among subjects

Variables	Severe Depression		P
	Absent (N = 177)	Present (N = 63)	
Age	62.18 ± 15.52	63.33 ± 15.09	0.611
Income ($)	404.40 ± 201.10	396.50 ± 184.83	0.798
MUFAs (g/day)	18.33 ± 6.08	16.53 ± 6.36	0.540
PUFAs (g/day)	16.22 ± 10.16	12.61 ± 9.67	0.014
SFAs (g/day)	17.77 ± 6.09	17.07 ± 6.13	0.432
TD F(g/day)	16.02 ± 6.60	13.11 ± 5.59	0.002
BMI (kg/m^2)^	28.54 ± 5.37	27.75 ± 5.10	0.298
WHR	0.99 ± 0.19	1.05 ± 0.20	0.032
Carbohydrates (g/day)	245.83 ± 63.49	220.12 ± 49.95	0.004
FBS (mg/dl)	111.80 ± 54.63	124.93 ± 72.02	0.135
SBP (mmHg)	143.14 ± 18.07	149.68 ± 17.04	0.011
DBP(mmHg)	94.33 ± 13.37	96.88 ± 13.44	0.196
TC (mg/dl)	196.55 ± 37.23	213.69 ± 33.96	0.001
LDL (mg/dl)	142.02 ± 26.35	148.39 ± 21.98	0.633
HDL (mg/dl)	60.81 ± 13.02	62.20 ± 16.17	0.346
TG (mg/dl)	160.63 ± 80.36	160.61 ± 66.16	0.999

Table 4 presents crude and adjusted data concerning severe depression associated with AMI. The results revealed that severe depression was independently associated with the risk of AMI (P = 0.025, OR = 2.6, 95% CI 1.1-5.8).

**Table 4:** AMI risk by depression before and after adjustment (N = 240)

Severe Depression	OR	95% C.I	p
Absent vs present (177/63)	3.1	1.7-5.7	0.000^a^
Absent vs present (177/63)	2.6	1.1-5.8	0.025^b^

^a^Before adjustment; ^b^Adjustment for other variables in unconditional binary logistic regression model controlling for anthropometrics parameter, lipid profile, socio-demographic status, and nutrient variables.

## Discussion

This study has demonstrated that severe depression symptoms are independently associated with AMI, after adjusting for other factors. In addition, lower total dietary fiber (TDF), carbohydrates, higher levels of total cholesterol (TC), a high waist-hip ratio (WHR) and high systolic blood pressure (SBP) were associated with individuals exhibiting severe depression symptoms.

After the war between Iran and Iraq between 1980 and 1988, psychological illness increased and it is reported that 60,000 suffer from such conditions [18]. There is a high incidence of suicide [19], believed to reflect the high levels of depression in this region. However, there may be reasons other than genetic factors and the war that cause depression in this population including poor living conditions and a lack of amenities for the general population [20].

Several studies have demonstrated that depression is associated with higher morbidity and mortality in patients with AMI [13,21-23]. In addition, patients who suffer from myocardial infarction (MI) commonly experience major depressive disorders [24]. For example, after adjusting for other risk factors, the risk of mortality for acute myocardial infarction is as much as four times higher because of major depression [25].

Mechanisms underlying the relationship between depression and CHD have yet to be elucidated. However, abnormal levels of biomedical coronary risk factors and neurohormonal dysregulation could explain why depression increases the occurrence of CHD or predicts its clinical outcome. Depressed subjects often exhibit more AMI risk factors including hypertension [26], and hyperlipidemia than those without depression [27].

Major depressive disorders can deregulate the autonomic nervous system (ANS) and the hypothalamic-pituitary-adrenal (HPA) axis, and this deregulation can result in elevated catecholamines and cortisol [21,23], The ANS down-regulation predisposes patients to myocardial ischemia owing to activated platelet factors [28], such as cytokines and growth factors. These factors accelerate atherosclerosis through the migration and adhesion of leukocytes, the promotion of oxidized LDL and free radicals [29].

Furthermore, HPA hyperactivity during depression alters the blood brain barrier by deregulation of the multidrug resistance p-glycoprotein (MRD PGP) [30]. Hyperactivity of MRD PGP induced by depression results in reduced transport of glucocorticoids to the brain, leading to glucocorticoid resistance [31]. It has been well documented that the discharge of corticosteroids caused by HPA hyperactivity in CVD patients leads to hypertension and hyperlipidemia [32]. Furthermore, a reduced heart rate and variability of this rate are more likely to be experienced by depressed subjects [33]. Such a reduction in the heart rate has been documented as a risk factor for cardiovascular diseases [34] including myocardial infarction and sudden cardiac death [35].

Inflammatory marker levels are higher than normal in depressed patients with CVD. One hypothesis is that depression itself promotes a mild inflammatory response, which unhealthy practices such as inactivity and cigarette smoking may foster, and leads to deregulation of hormonal systems (HPA) and increased susceptibility to infection. One very sensitive inflammatory marker, C-reactive protein (hsCRP), was shown by Kaptoge et al. [36] to be a specific risk factor for CVD. C-reactive protein is secreted by the liver, mainly in response to IL-1 and IL-6, which are pro-inflammatory cytokines [37]. Secretion of these cytokines is promoted by immune stimuli and an imbalance in them could be involved in the pathophysiology of depression. IL-6, a particularly potent stimulator of the HPA axis, is secreted as part of the response to stress. This could imply a cascade effect among physiological mechanisms and biomarkers, which acting individually or collectively might enhance depression and increase the risk for cardiovascular disease [38].

During depression there are increased levels of radical nitrogen species (RNS) and reactive oxygen species (ROS) such as peroxide, and the activities of pro-oxidant enzymes such as xanthine oxidase (XO) are also increased, nitric oxide (NO) levels are changed and there are indications of oxidative and nitrosative stress (O&NS) damage to fatty acids and DNA [39]. Peroxide circulates in the blood, and a recent case-control study by Maes et al. [40] showed that major depression patients have significantly higher plasma peroxide levels than normal individuals.

There is also strong evidence that depression increases oxidative damage to tissues [41,42]. Malondialdehyde (MDA), which can modify proteins and generate advanced lipoxidation end (ALE) products, is a by-product of polyunsaturated fatty acid peroxidation and is a widely-used indicator of oxidative stress. Its serum levels are significantly elevated in major depression [43]. ALEs are pro-inflammatory and therefore detrimental, weakening antioxidant defences and impairing DNA repair. They also have a key role in atherosclerosis and neurodegenerative disorders [44].

In the present study, polyunsaturated fatty acids (PUFAs) of those with severe depression were lower than in individuals with minor or moderate depression. Also, studies have demonstrated that a food frequency questionnaire (FFQ) can predict the concentration of PUFAs in the blood [45,46]. Several epidemiological [47] and case-control studies have demonstrated that a lower level of PUFAs is linked with depression [41,48-50] and CVD [51]. The mechanisms that affect PUFAs are unclear. However, one mechanism involves the regulation of the serotonergic [52] and dopaminergic systems [53]. Low levels of PUFAs predict low levels of 5-hydroxyindolacetic acid, a major metabolite of serotonin and an anti-depressant, in cerebrospinal fluid [54].

There are many functional interactions between dopaminergic systems and other systems including the serotoninergic, glutamatergic, GABAergic and cholinergic systems, which are related to behavioural processes [55]. PUFA deficiency deregulates the blood brain barrier [56], which is important for controlling mood disorders [57-59]. Moreover, major depression is associated with over-activity of the inflammatory response, affecting cytokines and alpha-interferon [60,61]. The pro-inflammatory series of eicosanoids are significantly affected by polyunsaturated fatty acids [62].

This study has demonstrated a significant association between severe depression and important dietary intake such as TDF and carbohydrates. A recent case-control study demonstrated significantly lower levels of dietary fiber in depressed patients [63]. Individuals who consume low fiber in their diets have low levels of many essential nutrients including carbohydrates and unsaturated fatty acids [64], and high levels of rapidly digested starches [65]. Dietary fiber affects depression itself and depression outcomes. It modulates serum lipids [66], blood pressure [67], inflammation [68], and waist-hip ratio (WHR) [69]. A diet rich in fiber also contains hundreds of phytochemicals [70], which protect neurons against injury by stimulating the production of several antioxidant enzymes, neurotrophic factors, protein chaperones and other proteins that provide neural protection against injury and disease [71].

There was a relationship between the level of severe depression and carbohydrate intake in the subjects. Carbohydrates influence mood and behavior [72,73], yet the mechanisms underlying such effects are unclear. However, carbohydrates may activate neurobiochemical pathways such as brain neurotransmitters that play a role in depression [57,58]. A diet rich in carbohydrates results in changes in blood glucose levels [74], stimulating the release of insulin. Insulin supplies cells with sugars and induces the transport of tryptophan (TRP) to the brain [75], by increasing plasma TRP levels compared to other large neutral amino acids (LNAAs) [57,58]. Therefore, carbohydrate intake induces an increase in the TRP/LNAA ratio, causing LNAAs to enter skeletal muscles [58,59].

It has been reported that the waist-hip ratio (WHR) is correlated with psychosocial factors [76], such as depression [77]. Furthermore, the body mass index (BMI) is weakly associated with depression, and abnormalities of the HPA axis and higher WHR is evident in patients suffering major depression [78].

Although several studies have documented that cigarette smoking is a risk factor in depressed patients [79,80]. We found no such correlation [49,81,82]. In this study, married subjects experienced fewer symptoms of depression, indicating that the presence of support affected depression in a positive manner [81,83]. There was no link between symptoms of depression and demographic factors such as age, sex, education or family history of CHD [84].

## Limitation of the study and conclusion

This case-control study investigated hospitalized AMI patients only and further studies are required to investigate an association between depression and AMI among non-hospitalized patients. The present study demonstrated that severe depression symptoms are independent risk factors for AMI. Furthermore, severe depression was associated with an unhealthy diet and AMI risk factors. On the basis of these findings, there are several recommendations for future research. First, validation of the present findings is required as this is the first study concerning the population in Ilam province. A larger sample size is required, particularly for the investigation of socio-demographic characteristics. Furthermore, new risk factors such as C-reactive protein, malondialdehyde (MDA) and homocysteine should be investigated in the Ilam province, as they have been linked with depression and the risk of AMI worldwide.

## Abbreviations

AMI: Acute myocardial infarction; ANS: autonomic nervous system; BMI: Body mass index; BP: Blood pressure; CES-D: Center for Epidemiologic Studies Depression Scale; CHD: Coronary heart disease; CVD: Cardiovascular vascular disease; DBP: Diastolic blood pressure; FBS: Fasting blood sugar; FFQ: Food frequency questionnaire; GABA: gamma-aminobutyric acid; HDL: High density lipoprotein; HPA: Hypothalamic-pituitary-adrenal; LDL: Low density lipoprotein; MRD PGP: Multidrug resistance p-glycoprotein; MUFA: Monounsaturated fatty acid; PUFA: Polyunsaturated fatty acids; SBP: Systolic blood pressure; SFA; Saturated fatty acids; TC: Total cholesterol; TDF: Total dietary fiber; TG: Triglyceride; WHR: Waist-hip ratio; $: Dollar.

## Competing interests

The authors declare that they have no competing interests.

## Authors' contributions

TY designed, collected the data, interpretation of the results and helped draft the manuscript. KS provided the patients and controls, assisted with interpretation of the results and helped draft the manuscript. FAR assisted in design and interpretation of the results. MK assisted with interpretation of the results. SA assisted with collected the data, interpretation of the results and helped draft the manuscript. SOJ provided the patients and controls, assisted with interpretation of the results. WTW was a co-supervisor for this study. GA assisted with collected the data, interpretation of the results. All authors read and approved the final manuscript.

## Authors' information

TY: Master Student in Nutritional Sciences at University Putra Malaysia. KS: Cardiologist and Lecture in Ilam Medical University and Head of the Mostafa Hospital, Iran. FAR and WTW: Cardiologist and Lecturer at University Putra Malaysia. MK: Nutritionist and Lecturer at University Putra Malaysia. SA: Master Student in Community Health Science. SJP: Orthopaedist at the Mostafa Hospital. GA is a nurse at the Mostafa Hospital.
